# Parents’ satisfaction of tele-rehabilitation for children with neurodevelopmental disabilities during the COVID-19 pandemic

**DOI:** 10.1186/s12875-022-01747-2

**Published:** 2022-06-07

**Authors:** Pamela Frigerio, Liliana Del Monte, Aurora Sotgiu, Costantino De Giacomo, Aglaia Vignoli

**Affiliations:** 1 Childhood and Adolescence Neurology and Psychiatry Unit, ASST GOM Niguarda, Piazza Ospedale Maggiore 3, Milan, 20162 Italy; 2 Maternal and Infantile Department of Pediatrics, ASST GOM Niguarda Milano, Piazza Ospedale Maggiore 3, Milan, 20162 Italy; 3 Health Sciences Department, Università Degli Studi Di Milano, Via di Rudinì 8, Milan, 20142 Italy

**Keywords:** Parents’ satisfaction, Tele-rehabilitation, COVID-19, Neurodevelopmental disabilities

## Abstract

**Background:**

The use of tele-rehabilitation in children was limited before the COVID-19 pandemic, due to culture, technology access, regulatory and reimbursement barriers.

**Methods:**

The study was conducted according to the CHERRIES (Checklist for reporting results of internet E-surveys) guidelines in order to provide quantitative and qualitative data about experience of patients with disabilities and their caregivers during Phase 1 of the COVID-19 pandemic, and their level of satisfaction. An online survey was developed using Google Forms and sent via email. The outcome measures were rated using a 5-point Likert Scale. Two additional open-ended questions were used to collect qualitative data.

**Results:**

One hundred twenty-eight out of 261 families responded to the survey: 80.5% of the caregivers reported they were satisfied with the tele-rehabilitation. More than a half (53%) of the families reported a high level of satisfaction with the involvement they received in defining and sharing of rehabilitation goals.

**Conclusions:**

The implementation of telehealth during the COVID-19 lockdown has allowed us to gain more information about the potential of tele-rehabilitation, and resulted in an excellent satisfaction of caregivers. With appropriate education and consistent models of care, an increased use of telehealth may provide advances in remote patient care.

**Trial registration:**

Not applicable.

**Supplementary Information:**

The online version contains supplementary material available at 10.1186/s12875-022-01747-2.

## Background

Following the spread of the COVID-19 outbreak in Italy in February 2020, strict measures have been implemented by the Italian Government. As a primary intervention to contain the SARS-CoV-2 infection spread, the Italian Government aimed at limiting the virus transmission by imposing a severe lockdown with daily-life restrictions and closure of educational, social, and manufacturing activities [1]. Lombardy has been one of the epicenters in Italy and one of the regions that have been most severely affected by the pandemic in Europe. Regional Health Authorities have recognized children and adolescents with neurological/psychiatric disorders as a priority and have authorized the continuation of services for “Childhood and Adolescence Neuropsychiatric Units”. They recommended interrupting standard and deferrable in-person rehabilitation therapies and/or integrating in-person rehabilitation sessions with remote therapy, to ensure continuity of care and to protect patients and healthcare professionals [2].

As a consequence, our Unit located at Niguarda Hospital in Milan (Italy) has been forced to implement rapid changes in the organization of rehabilitation care delivery, and new procedures have been developed in accordance with the national and regional emergency plans and recommendations. Since tele-rehabilitation was not included into our routine care, we rapidly updated and developed policies and procedures in our outpatient services according to the current laws on informed consent, data protection, and privacy. During this state of emergency declared by the Italian Government, the policy on reimbursement was also adjusted.

In-person sessions for outpatient children for whom neuropsychiatric assessment was ongoing were postponed. Therapy continued remotely for outpatient children with ongoing rehabilitation programs, when the Individual Rehabilitation Project (IRP) allowed so. Tele-rehabilitation was delivered through different modalities, namely phone contacts, video calls, online platforms, videos sent to the families, and other devices available for remote contact.

Although tele-rehabilitation has long been recognized as a promising means of providing rehabilitation therapy [3–7], the use of remote devices for rehabilitation in children was limited before the COVID-19 pandemic, due to culture, technology access, regulatory and reimbursement barriers [8, 9]. Now telemedicine is emerging as an indispensable resource to support the rehabilitation of patients and to ensure continuity of care. In keeping with the concept that this unprecedented period of healthcare crisis can also become an opportunity, we need to improve our ability to use innovative technologies to respond to the special healthcare needs of children with disabilities and their families [10–14]. To do this, we need to start to understand patients’ and caregivers’ perceptions, needs and levels of satisfaction.

The aim of this report is to provide quantitative and qualitative data about the level of satisfaction and experience by patients and caregivers during Phase 1 of the COVID-19 pandemic, when tele-rehabilitation emerged as the sole alternative to follow patients and to continue rehabilitation programs.

The secondary aim is to assess the association of caregivers’ and patients’ levels of satisfaction with other variables such as patients’ age, type of rehabilitation program, and device used.

## Methods

### Study design & setting

The study was conducted according to the CHERRIES (Checklist for reporting results of internet E-surveys) guidelines [15]. After reviewing the existing literature as well as other data sources or surveys with similar research topics, we structured an online survey containing 15 items to investigate general information and 18 specific items with multiple choices. The content of the survey is available in the supplemental material—appendix. The outcome measures were rated using a 5-point Likert Scale.

The questions were divided into a first part with general information and a second battery of 18 items divided into four sections: 1. tele-rehabilitation tools; 2. Communication and information; 3. Ability to develop and execute a treatment plan; 4. General patient satisfaction. Two additional open-ended questions were used to collect qualitative data, permitting detailed and more meaningful answers.

### Population

All caregivers of children receiving tele-rehabilitation during the lockdown period were eligible to participate. Inclusion criteria were an ongoing IRP and at least one remote contact during the lockdown period (February-June 2020) with any rehabilitation professional from our Unit of Child and Adolescence Neuropsychiatry, Mother and Child Department, Niguarda Hospital, in Milan, Italy. Rehabilitation professionals included: physiotherapists, speech therapists, occupational therapists, neuro-psychomotricist of the developmental age, professional educators, and psychiatric rehabilitation therapists.

### Study procedures

Caregivers were informed about the initiative by the therapist during their tele-rehabilitation session. Those who agreed to participate received an informative email with a link to the online survey. Since the online survey was anonymous and carried out with the CAWI (Computer Assisted Web Interviewing) method, completing the survey was considered a form of consent and no additional written consent was deemed necessary for this initiative by the ethics committee. Usability and technical functionality of the electronic questionnaire had been tested before sharing the questionnaire with the families.

The e-mail granted access to a single-use link to the survey, and was sent 20 weeks after the beginning of the remote intervention. Only one caregiver/parent was allowed to respond for each child.

We used an online survey tool through *Google Moduli* hosted on our Institution’s Communication and External Relations office that allows automated exporting of results.

### Measures

The outcome measures were rated using a 5-point Likert Scale. Results were collected in a dedicated database and exported to Microsoft Excel for validation and analyses.

### Statistical data analysis

Quantitative data were first submitted as customary descriptive statistics. Continuous variables were reported as mean ( ±) standard deviation or by median and range depending on the variable distribution, which was checked using the Shapiro–Wilk test. Various categorical variables were reported as relative and absolute frequency tables.

Statistical analysis was carried out using state/SE16.1.

Categorical variables were then checked by Fisher’s exact test, while logistic regression was used to verify the dependence of binary variables on continuous and categorical variables.

For qualitative data, our approach for the analysis is based on an inductive and phenomenological approach designed to gain the closest access possible to the parents’ experience. We conducted a summative content analysis to analyze survey responses inductively, which involves comparing contents and counting keywords, followed by interpretation of the context. This procedure has been already proposed [16]. It is composed of two stages:

1. Two researchers (L.D. and A.S.) worked independently to group similar responses into themes, where subthemes were constructed from repeating ideas. The researchers removed any responses deemed irrelevant (e.g., “N/A” or “none”).
2. After the 2 researchers came to a consensus on the themes and subthemes, a third researcher (P.F.) provided a check on the plausibility of themes and subthemes, and the consistency of analysis, adding to the rigorous approach [17]. Any discrepancies were then discussed as a group, where themes and subthemes were refined until agreement was reached. Keywords were then extracted and quantified using frequency counts [16].

This study was approved by the Institution’s Data Protection office and Communication and External Relations office, on behalf of the Ethic Committee (Comitato Etico Milano Area 3). The study complied with institutional regulations for anonymized studies and adhered to the principles of the Helsinki Declaration.

## Results

Between 6^th^ August, 2020 and 15^th^ October, 2020 we sent the link for completing the online survey to 261 families: 130 participated in the survey, and 128 responses were eligible for analysis.

Of the 128 responses, 99 (77.4%) were given by mothers, 27 (21%) by fathers and 2 (1.6%) by their relatives (1 foster parent and 1 grandmother).

Patients’ and parents/caregivers’ demographic data, patients’ ages and diagnosis are reported in Table 1 and Table 2, respectively.

**Table 1:** Patients’ and caregivers’ demographic data

**Patients**		**Caregivers/Parents**	
**Age (years)**	**n (%)**	**Age (years)**	**n (%)**
0–1^11/12^	6 (4.5)	< 25	3 (2.3)
2–3^11/12^	4 (3)	25–35	19 (15)
4–5^11/12^	28 (22)	36–45	63 (49.2)
6–10^11/12^	70 (55)	46–55	36 (28)
10–13^11/12^	14 (11)	56–65	7 (5.5)
> 14	6 (4.5)	> 65	0 (0)
**Caregivers’ educational level**		**n (%)**	
Primary school		4 (3)	
Secondary School		15 (12)	
High School		67 (52)	
University		42 (33)

**Table 2:** List of diagnostic groups in descending order

Diagnosis	n (%)
Mixed specific developmental disorders	49 (18.85)
Autism Spectrum Disorders	45 (17.31)
Genetic Syndromes	38 (14.62)
Behavioural and Emotional Disorders	34 (13.08)
Specific Language Disorder	32 (12.31)
Others	19 (7.3)
Specific developmental disorder of motor function	16 (6.15)
Specific Learning disorder	16 (6.15)
Cerebral Palsy	11 (4.23)

Proposal of remote rehabilitation was offered to 119 families (93% of respondents) for the first time during Phase 1 of the COVID-19 emergency.

Eleven (9%) patients received physiotherapy treatments, 12 (9%) educational intervention, 36 (28%) speech therapy, 29 (23%) psychomotor therapy, and 39 (31%) Equine-Assisted Therapy.

Although each intervention was based on the individual rehabilitation project, we can describe common goals for all intervention parent training and counselling.

In addition, we can list more common goals for each treatment:

- Speech therapy for improving receptive and expressive language skills, improving reading comprehension, reading speed and accuracy;
- Educational intervention for parent training for children with autism spectrum disorder;
- Neuropsicomotor Therapy for improving interaction and motor skills;
- Equestrian rehabilitation (which included occupational therapists, neuropsychomotor therapists and psychiatric rehabilitation therapists) for communications and interaction skills, improving fine motor abilities, narrative skills, and development of executive functions;
- Physiotherapy consisted of real-time treatment with children and with a parent and sharing of information and exercise programs to be implemented by the parents (parent training). Exercise did not follow a standardized scheme but was individualized for each patient based on his/her clinical features and type of device used.

Personalized video-tutorials made by physiotherapist were sent to parents, in which the exercises to be performed by the child were explained. The supervised tele-rehabilitation with physiotherapists allowed parents to be correctly guided and to maintain contact with the therapist.

Eighty-two households (64%) owned a personal computer (PC), 30 (23%) used a smartphone, and 16 (13%) a tablet.

Data about platforms used and level of satisfaction regarding these means are reported in Table 3.

**Table 3:** Platforms used and level of satisfaction

Platforms	n (%)	Satisfaction level n (%)				
		**Very satisfied**	**Satisfied**	**Partially satisfied**	**Dissatisfied**	**Very dissatisfied**
Skype	85 (66)	22 (26)	50 (59)	10 (12)	3 (3)	0
WhatsApp	10 (8)	2 (80)	1 (10)	1(10)	0	0
Meet	3 (2)	0	2 (33)	1(67)	0	0
Zoom	1(1)	0	1 (100)	0	0	0
Multiple platforms	16 (13)	5 (31)	10 (63)	1 (6)	0	0
No platforms	13 (10)	5 (38)	6 (46)	1 (8)	1 (8)	0

The whole cohort received parent–child educational sessions, 112 (88%) families received video calls, 12 (9%) were called through phone calls, and 4 (3%) were sent tools to perform the therapies (e.g. videos, drawings, pictures) between therapist and family.

In terms of management of computer tools, 86 (67%) patients were not independent. Therefore, 59 (46%) caregivers were asked to attend the entire session.

Data from the "Communication and Information" section (level of satisfaction with the information received, availability of staff, involvement and clarity of the language used by therapists) are reported in Table 4.

**Table 4:** Communication and Information section and level of satisfaction

Items	Satisfaction level n (%)				
	**Very satisfied**	**Satisfied**	**Partially satisfied**	**Dissatisfied**	**Very dissatisfied**
Information received by therapists	74 (58)	50 (39)	4 (3)	0	0
Availability of therapists with regard to the organization	87 (68)	35 (27)	6 (5)	0	0
Involvement in defining the activities	68 (53)	47 (37)	9 (7)	4 (3)	0
Clarity of the language used by therapists	80 (63)	44 (34)	4 (3)	0	0

Among caregivers, 39 (30.5%) were very satisfied with the tele-rehabilitation and 64 (50%) were satisfied, for a total of 80.5% of the caregivers (including very satisfied and satisfied) [95% Conf. Interval]. Conversely, 18 (14%) caregivers were partially satisfied and 7 (5.5%) dissatisfied. None of the caregivers reported that they were very dissatisfied.

Logistic regression showed no influence of the answer to the question *"*level of satisfaction of tele-rehabilitation" (Question 33) on the dichotomized answer to the question "caregiver’s participation in the therapy session*"* (Question 14) [likelihood ratio test: *p* = 0.1899], even though there was a statistically significant association between the dichotomized answer to Question number 33 and the dichotomized answer to Question number 14 (Fisher’s exact test: *p* = 0.048).

More than a half (68.5%) of the families reported a high level of satisfaction with the involvement received in the definition and sharing of rehabilitation goals, 47 (37%) stated that they were satisfied, 9 (7%) partially satisfied, 4 (3%) dissatisfied, 0 very dissatisfied.

Concerning the achievement of work objectives provided by the IRP, 42 (32%) families were very satisfied, 69 (53%) satisfied, 13 (10%) partially satisfied, 6 (5%) dissatisfied, 0 very dissatisfied.

Satisfaction with tele-rehabilitation in relation to the child’s ability to participate was rated as excellent by 70.3% of respondents [95% Conf. Interval].

Satisfaction degree of tele-rehabilitation in relation to the child’s ability to participate according to age groups and to type of rehabilitation program are reported in Table 5.

**Table 5:** Satisfaction degree of remote rehabilitation in relation to the child’s ability to participate analyzed by age groups and by type of rehabilitation program

Age (*years)*	n (%)	Satisfaction level n (%)				
		**Very satisfied**	**Satisfied**	**Partially satisfied**	**Unsatisfied**	**Very unsatisfied**
< 2	6 (4,5)	1 (17)	4 (67)	1 (17)	0	0
2–2,11	4 (3)	0	1 (25)	2 (50)	1 (25)	0
3–5,11	28 (22)	10 (36)	15 (53)	3 (11)	0	0
6–10,11	70 (55)	32 (46)	11 (15)	25 (36)	2 (3)	0
11–13,11	14 (11)	4 (29)	8 (57)	1 (7)	1 (7)	0
> 14 years	6 (4,5)	0	5 (83)	1 (17)	0	0
**Type of rehabilitation**	**n (%)**	**Satisfaction level** **n (%)**				
		**Very satisfied**	**Satisfied**	**Partially satisfied**	**Unsatisfied**	**Very unsatisfied**
Physiotherapy	11 (9)	3 (27)	6 (55)	1(9)	1 (9)	0
Speech therapy	36 (28)	16 (45)	16 (44)	3(8)	1 (3)	0
Educational Intervention	13 (10)	3 (23)	8 (61)	1(8)	1 (8)	0
Neuropsychomotor Therapy	30 (23)	7 (23)	14 (47)	9(30)	0	0
Therapeutic Riding	39 (30)	18 (46)	15 (38)	5(13)	1 (3)	0

The qualitative remarks at the end of the survey (open-ended questions) showed that many participants described the availability of the therapists as a positive aspect (30 families), as well as the continuity of the rehabilitation projects (20 families). Three caregivers expressed their desire to continue using video calls for meetings or therapies.

We obtained a total of 130 open-ended responses (79 responses to the first question on aspects most appreciated and 51 responses to the second question that addressed comments or suggestions). After removing irrelevant responses, 74 responses for the first question and 30 for the second were finally analyzed.

The analysis of the first question on aspects most appreciated generated 5 themes: *availability and competence of the therapists, continuity of treatment, involvement of caregivers, organizational aspects such as hours, flexibility, *etc*., revelation about feasibility and effectiveness*. The number of participants supporting each theme can be found in Table 6, with statement examples from participants.

**Table 6:** First open-ended response on aspects most appreciated. Number of answers for the question and for each of 5 themes (n), with some parent's statement examples

Aspects most appreciated (*n* = 74)
*Availability and professionalism of the therapists (n* = *30)*
⎯Availability of the therapist to modulate the sessions by meeting the ways and times of the child and the ability of the therapist, once the instrument has been set, to involve him and work with him independently without my continuous support, making him truly the protagonist of the session
⎯Availability and interest towards the child with the development of an alternative solution to attendance in a short time
⎯I appreciated the commitment of the educator to do everything possible to meet my son's problems. It was great to see the commitment of the health facilities and of those who work directly with our son to not be stopped by Lockdown, and to not leave us alone
*Continuity of treatment (n* = *25)*
⎯The continuity of the path (the sense of normality) despite the difficulties caused by lockdown
⎯The possibility for my daughter to continue a path with dedicated operators was useful and helpful through the long period of lockdown. Of course, nothing equates to face-to-face therapy
⎯My daughter managed to not "lose" her interest, affection and trust (which is fundamental) towards the psychomotor and the horse
*Involvement of caregivers (n* = *7)*
⎯It has allowed the therapist to 'get acquainted ' with the home context
⎯To be involved in my son's therapy
⎯I liked that they help my son very much and I also like that they communicate a lot with me about problems concerning my son
*Organizational aspects such as hours, flexibility, *etc*. (n* = *6)*
⎯Therapist's call before appointments
⎯Functionality and time schedule
⎯Flexibility of schedules without need of travelling
*Revelation about feasibility and effectiveness (n* = *6)*
⎯We never thought we would have such interesting results working on the pc, it was a well-exploited and well-structured opportunity
⎯My son could do the exercises in a family environment, I saw him more concentrated and less disoriented than when I went to the clinic
⎯The child manages to maintain attention even for 20 consecutive minutes (and it is a good result), what has always weighed the most on the child was the journey to accompany him inside Niguarda and take him back to school (more than once not it was collaborative)

The analysis of the second question that addressed comments or suggestions generated 3 themes: *technical aspects* (internet connection, web platform used, etc.), *propose tele-rehabilitation as a possible alternative, perhaps integrated with face-to-face therapy, claim that face-to-face therapy is irreplaceable*. The number of participants supporting each theme can be found in Table 7, with statement examples from participants.

**Table 7:** Second open-ended response addressed to comments or suggestions. Number of answer for the question and for each of 3 themes (n), with some parent's statement examples

Comments or suggestions (*n* = 30)
*Technical aspects (internet connection, web platform used, *etc*.) (n* = *9)*
⎯Create a platform for specific remote rehabilitation
⎯Identify a unique, cross-platform video conferencing tool so that all families use the same tool
⎯It would be desirable to use professional tools to ensure privacy on both sides; I do not consider the use of personal accounts of therapist entirely correct
*Propose tele-rehabilitation as a possible alternative, perhaps integrated with face-to-face therapy, n 2 above all for parent training. (n* = *6)*
⎯The meetings between therapists and family members could stay remote, it would be easier to manage the commitments!!
⎯I would like to be able to continue with this module, I find it winning, I see more progress from my son than the pre-lockdown mode
⎯We would like to continue with telerehabilitation (reducing the number of sessions in the presence)
*Claim that face-to-face therapy is irreplaceable (n* = *15)*
⎯Unfortunately, tele-rehabilitation can be good in a borderline condition like the one we experienced, nothing can replace the direct relationship between my son and the educator ⎯Tele-rehabilitation cannot replace the effectiveness and incisiveness of the direct relationship with the therapist whose praiseworthy support is appreciated
⎯In our opinion, although tele-rehabilitation has been excellent, it will not have to replace the current therapies without the lockdown

## Discussion

According to our experience and literature [18], children with neurodevelopmental disabilities and their families are at risk of developing psychological stress related to the COVID-19 pandemic and especially to the lockdown, which may determine the interruption of rehabilitation services [18]. After recognizing the needs of these fragile individuals, with the support of Regional Health Authorities, we have developed specific supportive services that can be provided even remotely. Since these tools have been supplied for the very first time during the pandemic in the great majority of patients, we thought that it would be particularly important to assess whether this new healthcare modality was responding to the patients’ needs, by evaluating parents’ satisfaction.

A particularly useful tool for the assessment of caregivers’ satisfaction is a questionnaire-based survey, because caregivers can answer questions anonymously and do not have to fear negative consequences because of their opinion. Moreover, self-administered questionnaires are—in contrast to interviews –cheaper, faster, and regarded as more voluntary [19].

Our study indicates that tele-rehabilitation of children with neurodevelopmental disabilities through tele-medicine is well accepted by the families. Indeed, 80.5% of them reported that they were satisfied with remote rehabilitation.

The possibility to continue treatment during the pandemic and the availability of therapists from remote were considered the main advantages.

These findings are in line with previous preliminary data [20–22], which suggest that an emergency program that provides continuity of care and support with tele-rehabilitation interventions may be beneficial for both the child’s and the parents’ well-being, with limited practical challenges.

Even if not directly assessed by the study tool, our direct experience during the pandemic seems to shows that tele-medicine in child neuropsychiatry produces positive effects for both the children and the families. On the child-side, we promoted and enabled continuity of care, maintained social contacts with their therapists, and reduced the risk of disrupting daily and weekly routines. Moreover, as extrapolations of this work, we can think that involving the parents in rehabilitation programs may improve the overall degree of satisfaction. Statistical analysis of data shows a statistically significant association between the dichotomized answer to Question “*"*level of satisfaction of remote rehabilitation" and the dichotomized answer to the question "caregiver’s participation in the therapy session*"* (Fisher’s exact test: *p* = 0.048); caregivers who had participated in the therapy session reported that they were satisfied with remote rehabilitation. Also, our experience seems to show that the parents’ role in facilitating the rehabilitation interventions is much more prominent using an online modality, as they can be more actively engaged by the therapists, thus increasing their self-esteem in parenting. In addition, according to our experience and literature, to be close to their own needs and those of their children, it seems to be useful to parents for both psychological and educational aspects [20].

The sample included in the present survey was highly representative of the usual customers of Italian Childhood and Adolescence Neurology and Psychiatry Units, and all the features of in-person rehabilitation that are usually offered have been covered remotely, although through innovative tools.

The development of specific rehabilitation tools created by the therapists has allowed to translate to remote also therapies where physical contact is very relevant. An example is that of Equine-Assisted Therapy, where the therapists made video calls from the Department and created specific videos to maintain goals about the ability to relate and on self-care, and realized interactive PowerPoint files dedicated to the horse theme was created to allow working on communication skills, attention, concentration, and alternation. Moreover, therapists of Equine-Assisted Therapy have implemented the use of Augmentative Alternative Communication (AAC). Indeed, customized materials have been developed, some horse-themed choosing activities, stories about horse’s daily routine and other materials have been translated into AAC, for children with special communicative needs. Based on the results of the survey, we can infer that the relationship with the horse combined with a careful reformulation of the proposals has allowed maintaining high motivation to treatment despite the distance. As a result, 84% of the families rated the Equine-Assisted Therapy positively with regard to the child’s ability to participate, and positive evaluations were recorded for other types of intervention as well.

The tools available to the families and the channels that were used were investigated in order to evaluate the accessibility to the tele-rehabilitation proposal: on-line platforms, e.g. Skype, were indicated as the most adequate instrument for rehabilitation purposes.

Considering that this was the first experience of tele-rehabilitation for almost the entire sample, we anticipated there would be some limits and critical issues. Therefore, we assessed data from Sect. 2 of the questionnaire (communication and information) to evaluate the effectiveness of the information received by the families.

In order to understand whether some treatments carry more issues or pitfalls than others and can therefore be more difficult to convert to tele-rehabilitation, we analyzed the data about the child’s ability to participate and the data about general satisfaction for each type of rehabilitation program. Some disciplines may be more challenging to be performed through tele-therapy (namely musculoskeletal work) and may require teaching of facilitation techniques to caregivers. In this view, the possibility to share previously recorded videos of rehabilitation sessions may help the families by supplying useful examples for continuity of care.

Moreover, children with behavioral or attention problems may face more difficulties following tele-rehabilitation sessions rather than face-to-face engagement.

One limitation of tele-rehabilitation that emerged from our survey is the child’s age, especially age younger than 3 years, even though the children in this age group were too few to permit further interpretation. As a matter of fact, toddlers need face-to-face rehabilitation treatments because of the importance of physical and relational contact. Indeed, they need treatments like physiotherapy or psychomotor therapy, where physical and relational contact is crucial.

Even though limited to the COVID-19-related lockdown, the findings of our survey indicate a positive response of caregivers of children with neurodevelopmental disabilities to tele-rehabilitation.

This result may encourage policy makers to implement these services into future healthcare models, recognizing the reduced costs of care and high patient/caregivers’ level of satisfaction. Indeed, considering the first positive response of caregivers of children with neurodevelopmental disabilities to tele-rehabilitation, during the course of the pandemic, Niguarda Hospital has implemented some tools for remote rehabilitation. The devices quota was increased (webcams, headphones and speakers), Zoom platform was installed on all computers (chosen as preferred method of communication for a company policy decision), and specific softwares for speech therapy rehabilitation in learning disabilities were purchased. For the future it could prove useful as remote rehabilitation improvement, a stable platform for rehabilitation interventions, telerehabilitation policies and procedures.

Furthermore, with regard to the reimbursement system, it is important to consider that despite the Italian Ministry of Health issued a document on 17 March 2014 to provide guidelines for Telehealth development [23], telemedicine in general and tele-rehabilitation in particular, were not expressly regulated by Italian law before the emergency due to Covid-19. So, until March 2020, in Italy there was no formal recognition and no reimbursement for clinical services such as Tele-rehabilitation.

During the emergency due to Covid-19, the Lombardy Region issued a circular to allow delivery of therapy remotely, by voice, video-call, video web platforms for all Childhood and Adolescence Neuropsychiatric services, for pediatric patients and their families, in accordance with the Decree of the General Director of the Welfare DG of Lombardy Region N. 2906 of the 8th of March and N. 3553 of the 15th of March 2020.

With this circular, during the pandemic emergency, tele-rehabilitation has been included in the list of reimbursed services and tariffs for these clinical services became comparable to those for the available usual care in presence.

For the purpose of regulating the methods used to carry out tele-rehabilitation, as a result of the pandemic, on 17 December 2020 the new National Guidelines for the Provision of Telemedicine Services were approved by the Permanent State Regions and Autonomous Provinces Conference. This document is playing a crucial role in the development of telemedicine in Italy because it aims to ensure consistent development of telemedicine throughout the country, going beyond the individual regional, hospital and services particularities. Thanks to this document entitled “National guidelines for the provision of telemedicine services,” drafted by the Ministry of Health, healthcare services provided remotely are officially a part of the National Healthcare Service and an updated version replacing the previous guidelines of 2014.

Italian regions must implement the Guidelines in their respective regional healthcare services. The Guidelines specify that the fee for a telemedicine service, tele-rehabilitation included, shall be equivalent to the fee for the same service when provided in the traditional way within the Basic Level of Assistance (so called LEA), including any cost-sharing. In this context, regions implemented telemedicine services within the Regional Healthcare Service, permitting a new method for providing health care, starting from tele-visits and limiting them to cases that involve ongoing care of patients who need ambulatory services and do not need to be examined.

### Limitations

Although the results of our study give some insights about the value of tele-rehabilitation for parents of children with neurodevelopmental disabilities, some limitations should be emphasized, concerning the heterogeneous nature of the types of treatments that can receive different feedback from the same family.

We are aware that the items and the questions identified for the questionnaire have been selected during the time of global lockdown, where the problems and the challenges were new for anyone. We tried to focus on the parents’ satisfaction about tele-rehabilitation, but we were unable to collect children’s opinions. So that, we cannot also exclude the possibility of missing relevant information to the remote care of children with neurodevelopmental disabilities. In order to capture the patient's satisfaction, for future research a useful approach can be to include in the questionnaire simple questions directed towards the children.

Another limitation of our study can be identified in the moment when the questionnaire was administered, 20 weeks after the beginning of the intervention, which can be considered a quite long period, but we preferred to obtain the parents’ assessment closed to the end of the tele-rehabilitation process, that usually lasts at least four-five months.

### Future research

Telerehabilitation seems to have an impact on financial and social stress factors for patients, and other different study projects could capture this data. New job perspectives can be assumed to reduce the dissatisfaction reported by some parents, it is important to consider new ways of tele-rehabilitation: for example using more performing platforms or mixed interventions "face to face"—remote. For future research, in order to fully assess the role of the parent in rehabilitation with and without a component of tele-rehabilitation, it would be better to have a study with two separate cohorts that compare levels of involvement and parental satisfaction.

## Conclusions

The usual care of children with special needs has become even more challenging during the COVID-19 lockdown, since parents found themselves involved throughout the whole day and often without the support of educational and rehabilitation services. To mitigate the feeling of being alone in direct caregiving of children with disabilities, we have developed potentially effective strategies to support the families of children with disabilities using tele-medicine approaches. This experience has allowed us to gain more information about the potentiality of tele-rehabilitation and resulted in an excellent level of satisfaction of the caregivers. With appropriate education and consistent models of care, an increased use of telehealth may provide advances in remote patient care.

## Supplementary Information

**Additional file 1.**

**Additional file 2.**

## Data Availability

The datasets generated and/or analysed during the current study are not publicly available due to limitations of ethical approval involving the patient data and anonymity but are available from the corresponding author on reasonable request.

## Abbreviations

IRP: Individual Rehabilitation Project
CAWI: Computer Assisted Web Interviewing
AAC: Augmentative Alternative Communication
