# Supplementary material for: Parents’ satisfaction of tele-rehabilitation for children with neurodevelopmental disabilities during the COVID-19 pandemic
Source: BMC Prim Care. 2022 Jun 7;23:146. doi: 10.1186/s12875-022-01747-2 (PMC9170348; doi:10.1186/s12875-022-01747-2)
Supplement: Supplementary file 1 — Additional file 1. [file 12875_2022_1747_MOESM1_ESM.docx]

***R3: telerehabilitation policies and procedures***

Despite the great advances made in the regulatory process of tele-medicine, services and regulation of tele-rehabilitation must be further developed. It is necessary to identify technological and digital solutions capable of ensuring a regular monitoring and continuity of care for patients by the Regional Health Service through tele-rehabilitation.

Tele-rehabilitation has shown some limitations related to the specificity of the rehabilitation field, for example it is difficult to evaluate new patients, establish a positive relationship, identify clinical symptoms, develop the program and aims of rehabilitation. In our study all patients had already started a rehabilitation program before the pandemic, and discontinued only because of restrictions for COVID-19. Therefore, in order to ensure the continuity of the rehabilitation program during the pandemic, tele-rehabilitation was quickly implemented without a specific training protocol.

Patients’ suitability for tele-rehabilitation and the choice of instruments for individual patients was based on the clinical reasoning of the therapist and the doctor in charge. Some feasibility criteria have been followed (such as the clinical evaluation of children and the availability of devices and connection), but without a specific protocol. This situation opened up two types of problems and responsibilities: the first relating to the professional competence of the therapist, who performed the therapy in order to ensure a honest, competent and responsible professional service, and the second related to the suitability of the equipement.

As for the equipment, the main limit was the lack of specific tools for tele-rehabilitation provided by the National Health Service (for example, software and health applications with the possibility of rehabilitation proposals executable on the computer) which could limit the possibility of offering tele-rehabilitation. Having specific rehabilitation platforms and software would make it possible to easily adapt proposals based on skills, patient needs and rehabilitation goals, and thus offer individualized programs.

We need platforms and software specifically created for rehabilitation with features defined by doctors and therapists. We have only one specific software that is dedicated to the rehabilitation of Specific Learning Disorders. It is necessary to have easy-to-use, low-cost platforms that are accessible to everyone, that safeguard privacy.

At present, most of the technological systems used have not been designed to carry out rehabilitative interventions, but have been adapted for this purpose. One example was the choice to use Skype as a free platform, despite the reduced ability to protect privacy and security. There is an urgent need to provide guidance on the choice of the most appropriate technological tool for the protection of patients' data in the context of tele-rehabilitation, in accordance with privacy provisions.

A further aspect to which attention has been paid is the acquisition of informed consent. Informed consent in tele-rehabilitation, especially for pediatric patients, must have the same basic requirements as traditional rehabilitation services.

During the emergency period, the national recommendations of the Istituto Superiore di Sanità in Italy (Report ISS COVID-19 n. 12/2020 - Interim indications for Telemedicine Assistance Services During the Emergency Health COVID-19. Version of 13 April 2020), allow you to use a simple reply by email as consent. It might be useful to implement the model of consent already in use with information on tele-rehabilitation, to explain in presence and in a clearer way the possible proposal of tele-rehabilitation, and at the same time acquire the signature of consent.

The current offer of tele-rehabilitation has evolved into a hybrid presence-remote system. The type of treatment, in presence or remotely, is carefully chosen based on the clinical evaluation of the patient
For face-to-face sessions, at the beginning of the session, the therapist must perform a triage keeping the social distance to investigate the main symptoms (e.g., body temperature > 37.5°C, cough, weakness) indicators of potential infection of COVID-19. In addition, the therapist must ask if the patient has been in contact with infected people in recent days. The same questions should be asked again at the beginning of each face-to-face session.

it is necessary to standardize the procedures and goals that characterize this therapeutic mode. Given the increasing burden of care and the need to provide adequate and continuous services to patients, tele-rehabilitation is becoming an interesting and promising model of care. Studies, protocols and guidelines on tele-rehabilitation are needed to understand the possibility to adapt the structures for the implementation of such rehabilitation services in order to share different experiences and better assess the cost-analysis of the effectiveness of this promising tool, potentially improving health services, especially during pandemics.
