# Supplementary material for: Parents’ satisfaction of tele-rehabilitation for children with neurodevelopmental disabilities during the COVID-19 pandemic
Source: BMC Prim Care. 2022 Jun 7;23:146. doi: 10.1186/s12875-022-01747-2 (PMC9170348; doi:10.1186/s12875-022-01747-2)
Supplement: Supplementary file 2 — Additional file 2. [file 12875_2022_1747_MOESM2_ESM.docx]

**SURVEY**

**PERCEIVED QUALITY**

**OF REMOTE REHABILITATION IN NEUROPSYCHIATRIC SERVICE OF CHILDHOOD AND ADOLESCENCE**

**GENERAL INFORMATIONS**

*(enter the data of the person completing the questionnaire)*

1. **What is your age?**

- < 25
- 25-35
- 36-45
- 46-55
- 56-65
- >65

1. **You have used our Neuropsychiatric Service of Childhood and Adolescence as:**

- Mother
- Father
- Other (specify)________________________

1. **What is your degree:**

- Elementary school licence
- Middle school licence
- High School diploma
- First or second level degree
- Other:…...

1. **What is your occupation:**

- Part Time employee
- Full Time employee
- Self employed
- Unemployed
- Retiree
- Other:….

1. **How many people were in your house during the lockdown period?**

(including yourself and your daughter/son, specify how many people lived in your house)

- 2
- 3
- 4
- 5
- More than 5

1. **How many children do you have?**

- 1
- 2
- More than 2

1. **How many of your children have followed a telerehabilitation program?**

- 1
- 2
- More than 2

1. **How old is your daughter/son?** (specify how old was your daughter/son when she/he was offered telerehabilitation)

- Less than 2
- 2-3
- 3-5
- 6-10
- 11-13
- 14 or more

1. **You and your daughter/son have used our service for** (specify even more than one option):

- [Neuropsychomotor](https://context.reverso.net/traduzione/inglese-italiano/Neuropsycomotor) Therapy [of the Developmental Age](https://context.reverso.net/traduzione/inglese-italiano/of+the+Developmental+Age)
- Educational intervention
- Psychological support intervention
- Assisted Therapy with horse
- Physiotherapy
- Other ______________________________

1. **Which type of intervention was suggested?** (it is possible to choose more than one answer)

- Video call therapist-child
- Video call therapist-parents
- Video call for group intervention between therapists and parents
- Video call for group intervention between therapists and children
- Call therapist-parents
- Sending of material from the therapist
- Sending of videos of the child performing the proposed activities
- Other ______________________________

1. **How many times a week in total did your child perform telerehabilitation?**

- 1
- 2
- 3
- >3

1. **Is your child able to independently manage the IT medium?**

- Yes
- No

1. **If not, are you the exclusive responsible for supporting your daughter/son in the use of the tool?** (Without help from the other parent or other family members)?

- Yes
- No

If not, who helped him/her? ___________________________.

1. **Has your participation been requested during the entire telerehabilitation session?**

- Yes
- No

1. **Before the lockdown period, had you already conduct activities in the mode of Telerehabilitation?**

(for example Parent training, Rehabilitation of Reading via online programs, etc.)

- Yes
- No

**ACCESSIBILITY OF THE TOOLS USED**

1. **With which types of devices could you use our remote service?**

(it is possible to choose more than one answer)

- Telephone
- PC
- Smartphone
- Other______________________

1. **Which platforms or methods did you use to use our service remotely?**
   (it is possible to choose more than one answer)
2. Zoom
3. Skype
4. Whatsapp video calls
5. Meet
6. Sending of material or video
7. Other ______________________

3. **To conduct the Telerehabilitation, did you use your devices or were they provided by the school, municipality, other institutions)?**

- Personal
- Provided by institutions and returned
- Provided by institutions and become personal
- Other____________________________

4. **How do you rate the proposed platforms of telerehabilitation in terms of their ease of use?** (meaning Skype, Zoom, Meet, Whatsapp Video calls, etc.)

- Very unsatisfactory
- Unsatisfactory
- Partially Satisfying
- Satisfying
- Very satisfying

5. **How do you assess the tools offered to conduct Telerehabilitation in terms of ease of use?** (meaning PC, Telephone, Tablet, IPad, etc.)

- Very unsatisfactory
- Unsatisfactory
- Partially Satisfying
- Satisfying
- Very satisfying

**6. How do you assess the working mode chosen in relation to the work objectives?**

- Very unsatisfactory
- Unsatisfactory
- Partially Satisfying
- Satisfying
- Very satisfying

**COMMUNICATION AND INFORMATION**

7. **How do you rate the clarity and completeness of the information received on the organisation of the treatment?** (Meaning the clarity about date and hour, the modality and the purpose of use of the offered materials)

- Very unsatisfactory
- Unsatisfactory
- Partially Satisfying
- Satisfying
- Very satisfying

1. **How do you assess the availability of staff in relation to the organization of telerehabilitation?** (meaning with the possibility to choose the easiest and most accessible tool, and the flexibility in agreeing on date and time)

- Very unsatisfactory
- Unsatisfactory
- Partially Satisfying
- Satisfying
- Very satisfying

1. **How do you evaluate your involvement by the operator in the definition and in sharing of the rehabilitation goals?**

- Very unsatisfactory
- Unsatisfactory
- Partially Satisfying
- Satisfying
- Very satisfying

1. **How do you assess your involvement by the operator in the definition of the activities to be carried out and in their implementation? (for example, did you choose together the more suitable activities to do in the home context, in relation to your needs?)**

- Very unsatisfactory
- Unsatisfactory
- Partially Satisfying
- Satisfying
- Very satisfying

1. **How do you assess the clarity of the staff in the language used to explain how they work? (meaning the ability to explain how to use materials, e.g. documents and games, or proposed tools, e.g. reading programs, Skype platform, etc.)**

- Very unsatisfactory
- Unsatisfactory
- Partially Satisfying
- Satisfying
- Very satisfying

1. **How do you assess in general the way in which telerehabilitation sessions have been delivered?**

- Very unsatisfactory
- Unsatisfactory
- Partially Satisfying
- Satisfying
- Very satisfying

**CHILD’S COMPLIANCE**

1. **How do you assess the telerehabilitation proposal received in relation to your son/daughter’s ability to participate?**

- Very unsatisfactory
- Unsatisfactory
- Partially Satisfying
- Satisfying
- Very satisfying

1. **How do you assess the structuring of time and materials in relation to your child’s attentive abilities?**

- Very unsatisfactory
- Unsatisfactory
- Partially Satisfying
- Satisfying
- Very satisfying

1. **How do you evaluate the proposal for remote rehabilitation in relation to the interest shown by your son/daughter?** (he/she participated with pleasure or had to be called several times to approach the instrument, he/she never asked to be able to "skip" the appointment, he/she abandoned the therapy session before the end)

- Very unsatisfactory
- Unsatisfactory
- Partially Satisfying
- Satisfying
- Very satisfying

**OVERALL SATISFACTION**

1. **How do you assess the telerehabilitation proposal received in relation to the work objectives of the Individualized Rehabilitation Project?**

- Very unsatisfactory
- Unsatisfactory
- Partially Satisfying
- Satisfying
- Very satisfying

1. **How do you evaluate telerehabilitation in terms of effective offer to meet your needs?** (thanks to the remote work you felt supported and perceived the proposed work as useful for your son/daughter)

- Very unsatisfactory
- Unsatisfactory
- Partially Satisfying
- Satisfying
- Very satisfying

1. **How do you assess the telerehabilitation offer?**

- Very unsatisfactory
- Unsatisfactory
- Partially Satisfying
- Satisfying
- Very satisfying

**DO YOU HAVE ANY COMMENTS OR SUGGESTIONS ON THIS SUBJECT? ____________________________________________________________________________________________________________________________________________________________________________________________________________________________________________________________________________________________________________________________________________________________________________________________**
